# Synthesis and Biological Properties of Novel Triazole-Thiol and Thiadiazole Derivatives of the 1,2,4-Triazole-3(5)-one Class

**DOI:** 10.3390/molecules19022199

**Published:** 2014-02-19

**Authors:** Esra Düğdü, Yasemin Ünver, Dilek Ünlüer, Kemal Sancak

**Affiliations:** Department of Chemistry, Karadeniz Technical University, Trabzon 61080, Turkey

**Keywords:** 1,2,4-triazole-5-one, 1,2,4-triazole-thiol, 1,3,4-thiadiazole, antioxidant and antimicrobial activities

## Abstract

2,2'-(4,4'(Butane-1,4-diyl/hexane-1,6-diyl)bis(3-methyl-5-oxo-4,5-dihydro-1,2,4-triazole-4,1-diyl)) diacetohydrazides **3a**,**b** were obtained via the formation of diethyl 2,2'-(4,4'(butane-1,4-diyl/hexane-1,6-diyl)bis(3-methyl-5-oxo-4,5-dihydro-1,2,4-triazole-4,1-diyl))diacetates **2a**,**b**, obtained starting from di-[3(methyl-2-yl-methyl)-4,5-dihydro-1*H*-[1,2,4]-triazole-5-one-4yl]-*n*-alkanes **1a**,**b** in two steps. The synthesis of the compounds **7a**,**b**–**9a**,**b** incorporating the 1,3,4-thiadiazole, and **10a**,**b**–**11a**,**b** with a 1,2,4-triazole-thiol nucleus as the second heterocycle, was performed by the acidic or basic treatment of compounds **4a**,**b**–**6a**,**b** which were obtained from the reaction of **3a**,**b** with several isothiocyanates. Newly synthesized compounds were screened for antimicrobial activities and their antioxidant properties by the 1,1-diphenyl-2-picryl hydrazyl (DPPH) radical scavenging method. Compounds **4a**,**b**, **5a**,**b**, **and** **6a**,**b** were found to possess good antioxidant properties. Almost all compounds have significant antimicrobial activities.

## 1. Introduction

Derivatives of 1,3,4-thiadiazoles and 1,2,4-triazole are known to exhibit anti-inflammatory, antiviral, analgesic, antimicrobial, anticonvulsant and antidepressant activity, the latter being usually explored by the forced swim test [1,2,3,4,5,6,7,8,9,10]. Among the pharmacological profiles of 1,3,4-thiadiazoles and 1,2,4-triazoles, their antimicrobial, anticonvulsant and antidepressant properties seem to be the best documented. Triazoles, in particular, substituted-1,2,4-triazoles and the open-chain thiosemicarbazide counterparts of 1,2,4-triazole, are among the various heterocycles that have received the most attention during the last two decades as potential antimicrobial agents [11]. Substitutions including thio-, alkylthio- and alkenylthio-derivatives have been carried out primarily at the 3-position of the 1,2,4-triazole ring, to afford potential antimicrobial agents that will overcome the abovementioned resistance problems.

Heterocycles containing a 1,2,4-triazole or 1,3,4-thiadiazole moiety, and the compounds consisting of 1,2,4-triazole and 1,3,4-thiadiazole condensed nucleus systems constitute a class of compounds possessing a wide spectrum of biological activities such as anti-inflammatory, antiviral and antimicrobial and antitumoral properties [12,13,14,15,16,17,18]. It was reported that more efficacious antibacterial compounds can be designed by joining two or more biologically active heterocyclic systems together in a single molecular framework. Keeping this observation in mind, this paper has presented the synthesis of new triazole thiadiazole derivatives incorporating different pharmacophores as hybrid molecules possessing antioxidant and antimicrobial activities [19].

## 2. Results and Discussion

**Scheme 1:**
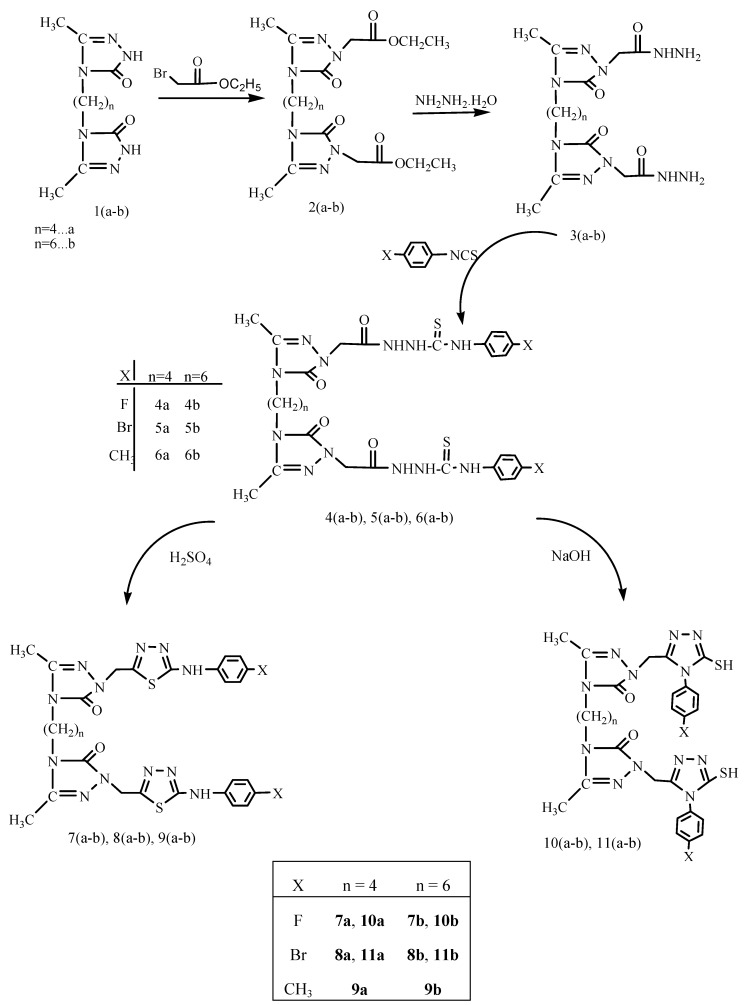
Synthetic pathway for the preparation of compounds **2**–**11**.

The synthesis of the intermediate and target compounds was performed according to the reactions outlined in Scheme 1. The starting compounds **1a**,**b** were prepared following a previously reported literature procedure [20]. The reaction of compounds **1a**,**b** with ethyl bromoacetate in the presence of sodium ethoxide produced diethyl 2,2'-(4,4'(butane-1,4-diyl/hexane-1,6-diyl)bis(3-methyl-5-oxo-4,5-dihydro-1,2,4-triazole-4,1-diyl))diacetates **2a**,**b**. The ethoxy group on compounds **2a**,**b** is an good leaving group for further nucleophilic substitution, thus reactions of **2a**,**b** with hydrazine hydrate converted these esters into the corresponding 2,2'-(4,4'(butane-1,4-diyl/hexane-1,6-diyl)bis(3-methyl-5-oxo-4,5-dihydro-1,2,4-triazole-4,1diyl))diacetohydrazides derivatives **3a**,**b** which were employed as key intermediates for synthesis of the target compounds.

Analytical and spectroscopic data of compounds **2a**,**b** confirmed this reaction by the additional signals derived from the –CH_2_CO_2_ Et group at the expected chemical shift values. Moreover, compounds **2a**,**b** gave a stable M+1 ion peak. The ^1^H-NMR spectra of compounds **3a**,**b** displayed no signals belonging to the -OCH_2_CH_3_ group; instead, new signals derived from the hydrazide structure appeared at 3.38–3.57 ppm (-NHNH_2_) and 9.16–9.17 ppm (-NHNH_2_) integrating for two protons and one proton, respectively (D_2_O exchange). Furthermore, compounds **3a**,**b** gave relatively stable M+1 ion peaks.

*N*-(4-Halo/methyl- phenyl)-2-(2-(4-(4-(1-(2-(2-(4-halo/methyl- phenylcarbamothioyl)hydrazinyl)-2-oxoethyl)-3-methyl-5-oxo-1*H*-1,2,4-triazole-4(5*H*)-yl)alkyl)-3-methyl-5-oxo-4,5-dihydro-1,2,4-triazole-1-yl)acetyl)hydrazinecarbothioamides **4a**,**b**–**6a**,**b** were obtained by the reaction of compounds **3a**,**b** with 4-fluorophenylisothiocyanate (**4**), 4-bromophenylisothiocyanate (**5**), or *p*-tolylisothiocyanate (**6**). The reaction was carried out at reflux temperature in ethanol and afforded the desired thiosemicarbazide derivatives, which were the starting materials for further cyclizations. The IR spectrum of compounds **4a**,**b**–**6a**,**b** displayed a broad signal at 3245 cm*^−^*^1^ due to three NH absorptions. In the ^1^H-NMR spectra of compounds **4a**,**b**–**6a**,**b**, these groups were observed at 9.56, 9.74, 10.24 ppm. Different from compound **3a**,**b**, the ^1^H- and ^13^C-NMR spectra of compounds **4a**,**b**–**6a**,**b** exhibited additional signals due to thiosemicarbazide moiety at the expected chemical shift values. In addition, compounds **4a**,**b**–**6a**,**b** gave relatively stable molecular ion peaks in the corresponding mass spectra.

The treatment of compounds **4a**,**b**–**6a**,**b** with cold concentrated sulfuric acid caused the conversion of the thiosemicarbazide structures into 1,3,4-thiadiazole rings; thus, 4,4'-(butane-1,4-diyl/hexane-1,6-diyl)bis(2-((5-(4-halogen/methylphenylamino)1,3,4-thiadiazole-2-yl)methyl)-5-methyl-2*H*-1,2,4-triazole-3(4*H*)-ones **7a**,**b**–**9a**,**b** were obtained. On the other hand, the cyclization of the same intermediates, **4a**,**b**–**6a**,**b** in the presence of 2 N NaOH produced 4,4'-(butane-1,4-diyl/hexane-1,6-diyl)bis(2-((4-(4-halogen/methylphenyl)-5-mercapto-4*H*-1,2,4-triazole-3-yl)methyl)-5-methyl-2*H*-1,2,4-triazole-3(4*H*)-ones **10a**,**b** and **11a**,**b**. In the IR spectra of compounds **7a**,**b**–**9a**,**b** the NH stretching band appeared at 3285–3198 cm^−1^. The NH proton resonated at about 10.20–10.27 ppm in the ^1^H-NMR spectra. The IR spectra of compounds **10a**,**b** and **11a**,**b** displayed SH stretching bands at 2962–2988 cm^−1^. Moreover, in the ^1^H-NMR spectra of compounds **10a**,**b** and **11a**,**b**, additional signals due to the SH group were observed at 13.88–13.99 ppm (D_2_O exchangeable), while the NH signals disappeared.

The newly synthesized compounds were screened for antioxidant properties by radical scavenging methods such as the 1,1-diphenyl-2-picryl hydrazyl (DPPH) assay. Compounds **4a**,**b**–**6a**,**b** possessing triazole-thiosemicarbazides have better antioxidant activity than **5a**,**b**. While compounds **4a**,**b** and **6a**,**b** have fluorine and methyl group substituents on the phenyl rings, compounds **5a**,**b** have bromine substituents. Thiosemicarbazide derivatives **4a**,**b**–**6a**,**b** also showed antibacterial activity against microorganisms. The 1,2,4-triazole nucleus is one of the active components present in many standard drugs and it is known to increase the pharmacological activity of the corresponding molecules. As an biological group, thiosemicarbazide groups in the triazole compounds should be considered for the synthesis of lead compounds in search of antioxidant and antimicrobial activity. All of the synthesized compounds were tested for antimicrobial activity. The antimicrobial screening suggests that among the newly synthesized compounds, **2a**, **3a**, **4a**,**b**–**11a**,**b** exhibited moderate activity against some of the tested microorganisms.

## 3. Experimental

### 3.1. General Information

Melting points were measured on an electrothermal apparatus and are uncorrected. ^1^H-NMR and ^13^C-NMR spectra were recorded on a Varian XL-200 NMR spectrophotometer (Palo Alto, CA, USA) in DMSO-*d_6_*. IR spectra were recorded on a Perkin-Elmer Spectrum one FT-IR spectrometer (Waltham, MA, USA) in KBr pellets. The MS spectra were measured with a Micromass Quattro LC/ULTIMA LC-MS/MS spectrometer (Waters, Milford, MA, USA) with EtOH as solvent. The experiment was performed in the positive ion mode. Elemental analyses were performed on a Hewlett-Packard 185 CHN analyzer. All the chemicals were obtained from Fluka Chemie AG (Buchs, Switzerland).

### 3.2. General Method for the Synthesis of Compounds **2**

The corresponding compound **1** (0.01 mol) was refluxed with an equivalent amount of sodium in absolute ethanol for 2 h. Then, ethyl bromoacetate (0.01 mol) was added and the mixture was refluxed for an additional 5 h. After evaporation of the solvent under reduced pressure at 35–40 °C, a solid appeared. This was recrystallized from 1:1 ethanol/water to afford the desired product.

*Diethyl 2,2'-(4,4'(butane-1,4-diyl)bis(3-methyl-5-oxo-4,5-dihydro-1,2,4-triazole-4,1-diyl))diacetate* (**2a**). Yield 78%, m.p. 150–151 °C. IR (υ, cm^−1^): 1,755 (ester C=O), 1,698 (triazole C=O), 1,638 (C=N), 1,212 (C-O); Anal. Calcd (%) for C_18_H_28_N_6_O_6_: C, 50.93; H, 6.65; N, 19.80. Found: C, 50.98; H, 6.68; N, 19.85; ^1^H-NMR (δ ppm): 1.14 (6H, t, *J* =7.0 Hz, 2 OCH_2_CH_3_), 1.52–1.59 (4H, bs, 2 NCH_2_CH_2_), 2.16 (6H, s, 2CH_3_), 3.57 (4H, t, 2 NCH_2_), 4.08 (4H, q, *J* = 7.0Hz, 2 OCH_2_CH_3_), 4.45 (4H, s, 2 NCH_2_C=O); ^13^C-NMR (δ ppm): 13.01 (2CH_3_), 19.94 (2OCH_2_CH_3_), 26.45 (2NCH_2_CH_2_), 43.79 (2 NCH_2_), 49.25 (2 NCH_2_C=O), 62.11 (2OCH_2_), 147.96 (2C=N), 159.71(2C=O), 169.74(2C=O hydrazide); MS (ESI): *m/z* (%) 425.21(M+1).

*Diethyl 2,2'-(4,4'(hexane-1,6-diyl)bis(3-methyl-5-oxo-4,5-dihydro-1,2,4-triazole-4,1-diyl))diacetate* (**2b**). Yield 80%, m.p. 142–143 °C. IR (υ, cm^−1^): 1748 (ester C=O), 1698 (triazole C=O), 1618 (C=N), 1208 (C-O); Anal. Calcd (%) for C_20_H_32_N_6_O_6_: C, 53.09; H, 7.13; N, 18.57. Found: C, 53.14; H, 7.17; N, 18.53; ^1^H-NMR (DMSO-*d_6_*, δ ppm): ^1^H-NMR (δ ppm): 1.20 (6H, bs, 2OCH_2_CH_3_), 1.52 (4H, bs, 2NCH_2_CH_2_), 2.16 (6H, s, 2CH_3_), 2.46 (4H, bs, 2NCH_2_CH_2_CH_2_), 4.09 (4H, bs, 2NCH_2_), 4.11 (4H, bs, 2OCH_2_CH_3_), 4.46 (4H, s, 2NCH_2_C=O); ^13^C-NMR (δ ppm): 11.06 (2CH_3_), 20.01 (2OCH_2_CH_3_), 25.58 (2NCH_2_CH_2_CH2), 27.75 (2NCH_2_CH_2_), 43.66 (2 NCH_2_), 49.26 (2 NCH_2_C=O), 62.15(OCH_2_), 146.28 (2C=N), 159.57 (2C=O), 169.72 (2C=O hydrazide); MS (ESI): *m/z* (%) 453.25 (M+1).

### 3.3. General Method for the Synthesis of Compounds **3**

A solution of the corresponding compound **2** (10 mmol) in *n*-butanol was refluxed with hydrazine hydrate (25 mmol) for 4 h. After cooling it to room temperature, a white solid appeared. This was recrystallized from 1:2 ethanol-water to obtain the desired compound.

*2,2'-(4,4'(Butane-1,4-diyl)bis(3-methyl-5-oxo-4,5-dihydro-1,2,4-triazole-4,1-diyl)) diacetohydrazide* (**3a**). Yield 72%, m.p. 204–206 °C. IR (υ, cm^−1^): 3203 and 3056 (NH-NH_2_), 1705 (triazole C=O), 1666 (hydrazide C=O); Anal. Calcd (%) for C_14_H_24_N_10_O_4_: C, 42.42; H, 6.10; N, 35.33. Found: C, 42.45; H, 6.17; N, 35.38; ^1^H-NMR (δ ppm): 1.54 (4H, bs, 2 NCH_2_CH_2_), 2.15 (6H, s, 2 CH_3_), 3.57–3.64 (8H, bs, 2 NCH_2 _+ 2 NHNH_2_), 4.16 (4H, s, 2 NCH_2_C=O), 9.16 (2H, s, 2NHNH_2_); ^13^C-NMR (δ ppm): 11.09 (2CH_3_), 26.24 (2NCH_2_CH_2_), 41.37 (2 NCH_2_), 46.76 (2 NCH_2_C=O), 144.33 (2C=N), 154.38 (2C=O), 170.12 (2C=O hydrazide); MS (ESI): *m/z* (%) 396.20 (M^+1^).

*2,2'-(4,4'(Hexane-1,6-diyl)bis(3-methyl-5-oxo-4,5-dihydro-1,2,4-triazole-4,1-diyl)) diacetohydrazide* (**3b**). Yield 73%, m.p. 232–233 °C. IR (υ, cm^−1^): 3213 and 3058 (NH-NH_2_), 1710 (triazole C=O), 1666 (hydrazide C=O); Anal. Calcd (%) for C_16_H_28_N_10_O_4_: C, 45.27; H, 6.65; N, 33.00. Found: C, 45.33; H, 6.72; N, 33.05; ^1^H-NMR (δ ppm): 1.26 (4H, bs, 2NCH_2_CH_2_CH_2_), 2.13 (6H, s, 2 CH_3_), 3.38–3.51 (8H, bs, 2 NCH_2 _+ 2 NHNH_2_), 4.17 (4H, s, 2 NCH_2_C=O), 9.17 (2H, s, 2NHNH_2_); ^13^C-NMR (δ ppm): 14.54 (2CH_3_), 20.37 (2NCH_2_CH_2_CH_2_), 28.47 (2NCH_2_CH_2_), 43.87 (2 NCH_2_), 49.35 (2 NCH_2_C=O), 146.87 (2C=N), 154.99 (2C=O), 171.45 (2C=O hydrazide); MS (ESI): *m/z* (%) 396.20 (M^+1^).

### 3.4. General Method for the Synthesis of Compounds **4**–**6**

A mixture of corresponding compound **3** (10 mmol) and 4-fluorophenylisothiocyanate (for compounds **4**), 4-bromophenylisothiocyanate (for compounds **5**) or *p*-tolylisothiocyanate (for compounds **6**) (15 mmol) was refluxed in ethanol for 4 h. The solution was cooled and a white solid appeared. This was filtered and recrystallized from ethanol to afford the desired product.

*N-(4-Fluorophenyl)-2-(2-(4-(4-(1-(2-(2-(4-fluorophenylcarbamothioyl)hydrazinyl)-2-oxoethyl)-3-methyl-5-oxo-1H-1,2,4-triazole-4(5H)-yl)butyl)-3-methyl-5-oxo-4,5-dihydro-1,2,4-triazole-1-yl)acetyl)hydrazinecarbothioamide* (**4a**). Yield 85%, m.p. 180–181 °C. IR (υ, cm^−1^): 3,245 (NH), 1,698 (triazole C=O), 1610 (C=N), 1191(C=S); Anal. Calcd (%) for C_28_H_32_F_2_N_12_O_4_S_2_: C, 47.85; H, 4.59; N, 23.92. Found: C, 47.89; H, 4.65; N, 23.97; ^1^H-NMR (δ ppm): 1.58 (4H, bs, 2 NCH_2_CH_2_), 2.47 (6H, s, 2 CH_3_), 3.56 (4H, t, 2 NCH_2_), 4.41 (4H, s, 2 NCH_2_C=O), 7.11–7.20 (4H, m, ArH), 7.33–7.40 (4H, m, ArH), 9.63 (2H, s, NH), 9.74 (2H, s, NH), 10.26 (2H, s, NH); ^13^C-NMR (δ ppm): 11.94 (2CH_3_), 26.17 (2NCH_2_CH_2_), 46.89 (2NCH_2_CH_2_), 56.71 (2NCH_2_C=O), ArC: [115.28 (2CH), 128.78 (2CH), 135.95 (2C), 144.47 (2C)], 154.66 (2C=N), 162.66 (2 triazole C=O), 167.32 (2 C=O), 181.61 (2C=S); MS (ESI): *m/z* (%) 703.20 (M+1).

*N-(4-Fluorophenyl)-2-(2-(4-(6-(1-(2-(2-(4-fluorophenylcarbonothioyl)hydrazinyl)-2-oxoethyl)-3-methyl-5-oxo-4,5-dihydro-1,2,4-triazole-4(5H)-yl)hexyl)-3-methyl-5-oxo-4,5-dihydro-1,2,4-triazole-1-yl)acetyl)hydrazinecarbothioamide* (**4b**). Yield 80%, m.p. 170–171 °C. IR (υ, cm^−1^): 3234 (NH), 1688 (triazole C=O), 1610 (C=N), 1189 (C=S); Anal. Calcd (%) for C_30_H_36_F_2_N_12_O_4_S_2_: C, 49.30; H, 4.97; N, 23.00. Found: C, 49.36; H, 4.92; N, 23.07; ^1^H-NMR (δ ppm): 1.15 (4H, bs, 2 NCH_2_CH_2_CH_2_), 1.51 (4H, bs, 2 NCH_2_CH_2_), 2.14 (6H, s, 2 CH_3_), 3.52 (4H, bs, 2 NCH_2_), 4.02 (4H, bs, 2 NCH_2_C=O), 6.76–7.45 (8H, m, ArH), 9.58 (2H, s, NH), 9.81 (2H, s, NH), 10.74 (2H, s, NH); ^13^C-NMR (δ ppm): 11.86 (CH_3_), 20.45 (2NCH_2_CH_2_CH_2_), 27.19 (NCH_2_CH_2_), 45.93 (NCH_2_CH_2_), 55.78 (NCH_2_C=O), ArC: [115.72 (2CH), 128.95 (2CH), 136.05 (2C), 144.90 (2C)], 154.86 (2C=N), 162.68 (2 triazole C=O), 168.60 (2 C=O), 181.64 (2C=S); MS (ESI): *m/z* (%) 730.24 (M+1).

*N-(4-Bromophenyl)-2-(2-(4-(4-(1-(2-(2-(4-bromophenylcarbamothioyl)hydrazinyl)-2-oxoethyl)-3-methyl-5-oxo-1H-1,2,4-triazole-4(5H)-yl)butyl)-3-methyl-5-oxo-4,5-dihydro-1,2,4-triazole-1-yl)-acetyl)hydrazinecarbothioamide* (**5a**). Yield 76%, m.p. 206–207 °C. IR (υ, cm^−1^): 3265 (NH), 1705 (triazole C=O), 1587 (C=N), 1192 (C=S); Anal. Calcd (%) for C_28_H_32_Br_2_N_12_O_4_S_2_: C, 40.78; H, 3.91; N, 20.38. Found: C, 40.75; H, 3.98; N, 20.42; ^1^H-NMR (δ ppm):1.04 (4H, bs, 2 NCH_2_CH_2_), 2.16 (6H, s, 2 CH_3_), 3.57 (4H, t, 2 NCH_2_), 4.43 (4H, s, 2 NCH_2_C=O), 7.38–7.55 (8H, m, ArH), 9.67 (2H, s, 2NH), 9.83 (2H, s, 2NH), 10.30 (2H, s, 2NH); ^13^C-NMR (δ ppm): 11.25 (CH_3_), 25.45 (NCH_2_CH_2_), 46.37(NCH_2_CH_2_), 56.79 (NCH_2_C=O), ArC: [115.34 (2CH), 128.86 (2CH), 135.98 (2C), 144.46 (2C)], 154.67 (2C=N), 162.69 (2 triazole C=O), 167.35 (2 C=O), 181.62 (2 C=S); MS (ESI): *m/z* (%) 824.05 (M^+^).

*N-(4-Bromophenyl)-2-(2-(4-(6-(1-(2-(2-(4-bromophenylcarbonothioyl)hydrazinyl)-2-oxoethyl)-3-methyl-5-oxo-4,5-dihydro-1,2,4-triazole-4(5H)-yl)hexyl)-3-methyl-5-oxo-4,5-dihydro-1,2,4-triazole-1-yl)acetyl)hydrazinecarbothioamide* (**5b**). Yield 79%, m.p. 190–191 °C. IR (υ, cm^−1^): 3224 (NH), 1711 (triazole C=O), 1668 (hydrazide C=O), 1185 (C=S); Anal. Calcd (%) for C_30_H_36_Br_2_N_12_O_4_S_2_: C, 42.26; H, 4.26; N, 19.71. Found: C, 42.21; H, 4.30; N, 19.76; ^1^H-NMR (δ ppm): 1.27 (4H, bs, 2 NCH_2_CH_2_CH_2_), 1.56 (4H, bs, 2 NCH_2_CH_2_), 2.54 (6H, s, 2 CH_3_), 3.60 (4H, t, 2 NCH_2_), 4.44 (4H, s, 2 NCH_2_C=O), 7.10–7.25 (4H, m, ArH), 7.38–7.49 (4H, m, ArH), 9.66 (2H, s, 2NH), 9.76 (2H, s, 2NH), 10.29 (2H, s, 2NH); ^13^C-NMR (δ ppm): 11.94 (2CH_3_), 20.50 (2NCH_2_CH_2_CH_2_), 26.16 (2NCH_2_CH_2_), 46.80 (2NCH_2_CH_2_), 56.72 (2NCH_2_C=O), ArC: [115.27 (2CH), 128.74 (2CH), 135.90 (2C), 144.41 (2C)], 154.61 (2C=N), 162.68 (2 triazole C=O), 168.35 (2C=O), 181.64 (2C=S); MS (ESI): *m/z* (%) 852.08 (M^+^).

*N-(p-Tolyl))-2-(2-(3-methyl-4-(4-(3-methyl-5-oxo-1-(2-oxo-2-(2-(p-tolylcarbamothioyl)hydrazinyl)-ethyl)-1H-1,2,4-triazole-4(5H)-yl)butyl)-5-oxo-4,5-dihydro-1,2,4-triazole-1-yl)acetyl)hydrazine-carbothioamide* (**6a**). Yield 77%, m.p. 218–219 °C. IR (υ, cm^−1^): 3224 (NH), 1705 (triazole C=O), 1637 (hydrazide C=O), 1188 (C=S); Anal. Calcd (%) for C_30_H_38_N_12_O_4_S_2_: C, 51.86; H, 5.51; N, 24.19. Found: C, 51.90; H, 5.57; N, 24.11; ^1^H-NMR (δ ppm):1.58 (4H, bs, 2 NCH_2_CH_2_), 2.15 (6H, s, 2 CH_3_), 2.27 (6H, s, 2 phenyl-CH_3_), 3.39 (4H, t, 2 NCH_2_), 4.42 (4H, s, 2 NCH_2_C=O), 7.14–7.23 (8H, m, ArH), 9.56 (2H, s, 2NH), 9.65 (2H, s, 2NH), 10.24 (2H, s, 2NH); ^13^C-NMR (δ ppm): 11.94 (CH_3_), 21.09 (2 phenyl-CH_3_), 26.17 (NCH_2_CH_2_), 46.93 (NCH_2_CH_2_), 58.62 (NCH_2_C=O), ArC: [120.54 (2CH), 129.27 (2CH), 137.07 (2C), 144.47 (2C)], 154.64 (2C=N), 167.31 (2 triazole C=O), 167.35 (2 C=O), 181.34 (2C=S); MS (ESI): *m/z* (%) 694.26(M^+^).

*N-(p-Tolyl))-2-(2-(3-methyl-4-(4-(3-methyl-5-oxo-1-(2-oxo-2-(2-(p-tolylcarbamothioyl)hydrazinyl)-ethyl)-1H-1,2,4-triazole-4(5H)-yl)hexyl)-5-oxo-4,5-dihydro-1,2,4-triazole-1-yl)acetyl)hydrazine-carbothioamide* (**6b**). Yield 76%, m.p. 185–186 °C. IR (υ, cm^−1^): 3247 (NH), 1688 (triazole C=O), 1590 (C=N), 1190 (C=S); Anal. Calcd (%) for C_32_H_42_N_12_O_4_S_2_: C, 53.17; H, 5.86; N, 23.25. Found: C, 53.22 H, 5.92; N, 23.48; ^1^H-NMR (δ ppm): 1.26 (4H, bs, 2NCH_2_CH_2_CH_2_), 1.54 (4H, bs, 2 NCH_2_CH_2_), 2.17 (6H, s, 2CH_3_), 2.29 (6H, s, 2 phenyl-CH_3_), 3.51 (4H, t, 2 NCH_2_), 4.35 (4H, s, 2 NCH_2_C=O), 7.07–7.13 (4H, m, ArH), 7.41–7.64 (4H, m, ArH), 9.61 (2H, s, 2 NH), 9.80 (2H, s, 2NH), 10.35 (2H, s, 2NH); ^13^C-NMR (δ ppm): 12.86 (CH_3_), 20.95 (2NCH_2_CH_2_CH_2_), 21.00 (2 phenyl-CH_3_), 25.58 (NCH_2_CH_2_), 43.25 (NCH_2_CH_2_), 56.12 (NCH_2_C=O), ArC: [120.67 (2CH), 127.65 (2CH), 138.76 (2C), 145.35 (2C)], 153.50 (2C=N), 160.21 (2 triazole C=O), 169.67 (2C=O), 180.81 (2C=S); MS (ESI): *m/z* (%) 722.29(M^+^).

### 3.5. General Method for the Synthesis of Compounds **7**–**9**

A mixture of corresponding thiosemicarbazides **4**–**6** (10 mmol) in cold concentrated sulfuric acid (30 mL) was stirred for 10 min then, the mixture was allowed to reach room temperature. After stirring for an additional 30 min, the resulting solution was poured into ice cold water and made alkaline to pH 8 with ammonia. The precipitated product was filtered, washed with water and recrystallized from ethanol to afford the pure compounds.

*4,4'-(Butane-1,4-diyl)bis(2-((5-(4-fluorophenylamino)1,3,4-thiadiazole-2-yl)methyl)-5-methyl-2H-1,2,4-triazole-3(4H)-one)* (**7a**). Yield 88%, m.p. 300–301 °C. IR (υ, cm^−1^): 3267 (NH), 1694 (triazole C=O), 1621 (C=N); Anal. Calcd (%) for C_28_H_28_F_2_N_12_O_2_S_2_: C, 50.44; H, 4.23; N, 25.21. Found: C, 50.49; H, 4.18; N, 25.28; ^1^H-NMR (δ ppm): 1.54 (4H, bs, NCH_2_CH_2_), 2.16 (6H, s, 2CH_3_), 3.58 (4H, bs, 2NCH_2_CH_2_), 5.07 (4H, s, CH_2_), 7.11–7.19 (4H, m, ArH), 7.54–7.61 (4H, m, ArH), 10.20 (2H, s, 2NH); ^13^C-NMR (δ ppm): 11.98 (2CH_3_), 26.10 (2NCH_2_CH_2_), 38.84–41.34 (DMSO-*d_6_*+2NCH_2_CH_2_), 44.03 (2 CH_2_), ArC: [105.00 (2C), 116.58 (2CH), 119.89 (2CH), 137.61 (2C), 145.25 (2C), 153.77 (2C)], 155.57 (2 C=N), 160.32 (2 triazole C=O), MS (ESI): *m/z* (%) 666.19 (M^+^).

*4,4'-(Hexane-1,6-diyl)bis(2-((5-(4-fluorophenylamino)1,3,4-thiadiazole-2-yl)methyl)-5-methyl-2H-1,2,4-triazole-3(4H)-one)* (**7b**). Yield 85%, m.p. 278–279 °C. IR (υ, cm^−1^): 3219 (NH), 1691 (triazole C=O), 1,627 (C=N); Anal. Calcd (%) for C_30_H_32_F_2_N_12_O_2_S_2_: C, 51.86; H, 4.64; N, 24.19. Found: C, 51.82; H, 4.68; N, 24.12; ^1^H-NMR (δ ppm): 1.20 (4H, bs, 2NCH_2_CH_2_CH_2_), 1.58 (4H, bs, NCH_2_CH_2_), 2.21 (6H, s, 2CH_3_), 3.48 (4H, bs, 2NCH_2_CH_2_), 5.11 (4H, s, 2CH_2_), 7.12–7.20 (8H, m, ArH), 10.26 (2H, s, 2NH); ^13^C-NMR (δ ppm): 11.25 (2CH_3_), 20.98 (2NCH_2_CH_2_CH_2_), 26.32 (2NCH_2_CH_2_), 38.80–41.41 (DMSO-*d_6_*+2NCH_2_CH_2_), 44.56 (2CH_2_), ArC: [101.07 (2C), 117.45 (2CH), 120.24 (2CH), 137.65 (2C), 145.20 (2C), 153.76 (2C)], 155.51 (2C=N), 160.30 (2 triazole C=O), MS (ESI): *m/z* (%) 694.22 (M^+^).

*4,4'-(Butane-1,4-diyl)bis(2-((5-(4-bromophenylamino)1,3,4-thiadiazole-2-yl)methyl)-5-methyl-2H-1,2,4-triazole-3(4H)-one)* (**8a**). Yield 83%, m.p. 299–300 °C. IR (υ, cm^−1^): 3241 (NH), 1690 (triazole C=O), 1,603 (C=N); Anal. Calcd (%) for C_28_H_28_Br_2_N_12_O_2_S_2_: C, 43.40; H, 3.77; N, 20.94. Found: C, 43.46; H, 3.79; N, 20.98; ^1^H-NMR (δ ppm):1.54 (4H, bs, NCH_2_CH_2_), 2.16 (6H, s, 2CH_3_), 3.58 (4H, bs, 2NCH_2_CH_2_), 5.07 (4H, s, CH_2_), 7.11–7.19 (8H, m, ArH), 10.23 (2H, s, 2NH); ^13^C-NMR (δ ppm): 11.19 (2CH_3_), 25.31 (2NCH_2_CH_2_), 38.84–41.31 (DMSO-*d_6_*+2NCH_2_CH_2_), 43.22 (2CH_2_), ArC: [113.11 (2C), 119.21 (2CH), 131.69 (2CH), 139.59 (2C), 144.43 (2C), 152.96 (2C)], 155.02 (2C=N), 164.85 (2 triazole C=O), MS (ESI): *m/z* (%) 788.02 (M^+^).

*4,4'-(Hexane-1,6-diyl)bis(2-((5-(4-bromophenylamino)1,3,4-thiadiazole-2-yl)methyl)-5-methyl-2H-1,2,4-triazole-3(4H)-one)* (**8b**). Yield 80%, m.p. 243–244 °C. IR (υ, cm^−1^): 3285 (NH), 1703 (triazole C=O), 1667 (C=N); Anal. Calcd (%) for C_30_H_32_Br_2_N_12_O_2_S_2_: C, 44.12; H, 3.95; N, 20.58. Found: C, 44.19; H, 4.05; N, 20.62; ^1^H-NMR (δ ppm): 1.24 (4H, bs, 2NCH_2_CH_2_CH_2_), 1.50 (4H, bs, NCH_2_CH_2_), 2.22 (6H, s, 2CH_3_), 3.45 (4H, bs, 2NCH_2_CH_2_), 5.10 (4H, s, 2CH_2_), 7.11–7.20 (8H, m, ArH), 10.28 (2H, s, 2NH); ^13^C-NMR (δ ppm): 11.02 (2CH_3_), 20.96 (2NCH_2_CH_2_CH_2_), 24.98 (2NCH_2_CH_2_), 38.84–41.36 (DMSO-*d_6_*+2NCH_2_CH_2_), 43.19 (2CH_2_), ArC: [115.15 (2C), 120.11 (2CH), 131.60 (2CH), 139.50 (2C), 144.35 (2C), 150.55 (2C)], 154.80 (2C=N), 164.80 (2 triazole C=O) MS (ESI): *m/z* (%) 817.07 (M+1).

*4,4'-(Butane-1,4-diyl)bis(2-((5-(p-toluidino)1,3,4-thiadiazole-2-yl)methyl)-5-methyl-2H-1,2,4-triazole-3(4H)-one)* (**9a**). Yield 76%, m.p. 303–304 °C. IR (υ, cm^−1^): 3240 (NH), 1692 (triazole C=O), 1645 (C=N); Anal. Calcd (%) for C_30_H_34_N_12_O_2_S_2_: C, 54.69; H, 5.20; N, 25.51. Found: C, 54.60; H, 5.27; N, 25.58; ^1^H-NMR (δ ppm): 1.54 (4H, bs, NCH_2_CH_2_), 2.18 (6H, s, 2CH_3_), 2.23 (6H, s, 2 phenyl-CH_3_), 3.59 (4H, bs, 2NCH_2_CH_2_), 5.07 (4H, s, 2CH_2_), 7.13–7.45 (8H, m, ArH), 10.24 (2H, s, 2NH); ^13^C-NMR (δ ppm): 12.00 (2CH_3_), 21.03 (2 phenyl-CH_3_), 26.14 (2NCH_2_CH_2_), 38.84–41.35 (DMSO-*d_6_*+2NCH_2_CH_2_), 44.07 (2CH_2_), ArC: [106.27 (2C), 129.74 (2CH), 130.20 (CH), 138.83 (2C), 145.20 (2C), 153.54 (2C)], 154.96 (2C=N), 166.33 (2 triazole C=O), MS (ESI): *m/z* (%) 658.24 (M^+^).

*4,4'-(Hexane-1,6-diyl)bis(2-((5-(p-toluidino)1,3,4-thiadiazole-2-yl)methyl)-5-methyl-2H-1,2,4-triazole-3(4H)-one)* (**9b**). Yield 77%, m.p. 269–270 °C. IR (υ, cm^−1^): 3198 (NH), 1690 (triazole C=O), 1656 (C=N); Anal. Calcd (%) for C_32_H_38_N_12_O_2_S_2_: C, 55.96; H, 5.58; N, 24.47. Found: C, 56.04; H, 5.68; N, 24.40 ^1^H-NMR (δ ppm): 1.24 (4H, bs, 2NCH_2_CH_2_CH_2_), 2.25 (6H, s, 2 phenyl-CH_3_), 1.50 (4H, bs, NCH_2_CH_2_), 2.23 (6H, s, 2CH_3_), 3.49 (4H, bs, 2NCH_2_CH_2_), 5.15 (4H, s, 2CH_2_), 7.14–7.25 (8H, m, ArH), 10.27 (2H, s, 2NH);^ 13^C-NMR (δ ppm): 12.01 (2CH_3_), 20.94 (2NCH_2_CH_2_CH_2_), 21.03 (2 phenyl-CH_3_), 26.18 (2NCH_2_CH_2_), 38.80–41.39 (DMSO-*d_6_*+2NCH_2_CH_2_), 44.07 (2CH_2_), ArC: [108.27 (2C), 128.79 (2CH), 130.21 (CH), 138.73 (2C), 145.28 (2C), 150.05 (2C)], 154.00 (2C=N), 167.56 (2 triazole C=O), MS (ESI): *m/z* (%) 687.27 (M+1).

### 3.6. General Method for the Synthesis of Compounds **10** and **11**

A solution of corresponding carbothioamide (**4**–**6**) (10 mmol) inequivalent amount of 2 N NaOH solution was refluxed for 3 h. The resulting solution was cooled to room temperature and acidified topH 3–4 with 37% HCl. The precipitate formed was filtered, washedwith water and recrystallized from dimethyl sulfoxide/water (1:1) to afford the desired compound.

*4,4'-(Butane-1,4-diyl)bis(2-((4-(4-fluorophenyl)-5-mercapto-4H-1,2,4-triazole-3-yl)methyl)-5-methyl-2H-1,2,4-triazole-3(4H)-one)* (**10a**). Yield 89%, m.p. 304–305 °C. IR (υ, cm^−1^): 2962 (SH), 1676 (triazole C=O), 1589 (C=N); Anal. Calcd (%) for C_28_H_28_F_2_N_12_O_2_S_2_: C, 50.44; H, 4.23; N, 25.21. Found: C, 50.48; H, 4.27; N, 25.28; ^1^H-NMR (δ ppm): 1.27 (4H, bs, NCH_2_CH_2_), 2.05 (6H, s, 2CH_3_), 3.46 (4H, bs, 2NCH_2_CH_2_), 4.79 (4H, s, 2CH_2_), 7.23–7.26 (8H, m, 2ArH), 13.95 (2H, s, 2SH); ^13^C-NMR (δ ppm): 11.71 (2CH_3_), 26.08 (2NCH_2_CH_2_), 38.82–41.31 (DMSO-d_6_+2NCH_2_CH^2^+2CH_2_), ArC: [116.62 (2C), 129.84 (2C), 130.43 (CH) 145.04 (2CH), 148.54 (2C), 153.08 (2C)], 165.32 (2C=N), 169.27 (2 triazole C=O), MS (ESI): *m/z* (%) 667.19 (M^+^).

*4,4'-(Hexane-1,6-diyl)bis(2-((4-(4-fluorophenyl)-5-mercapto-4H-1,2,4-triazole-3-yl)methyl)-5-methyl-2H-1,2,4-triazole-3(4H)-one)* (**10b**). Yield 81%, m.p. 310–311 °C. IR (υ, cm^−1^): 2988 (SH), 1678 (triazole C=O), 1580 (C=N); Anal. Calcd (%) for C_30_H_32_F_2_N_12_O_2_S_2_: C, 51.86; H, 4.64; N, 24.19. Found: C, 51.82; H, 4.72; N, 24.10; ^1^H-NMR (δ ppm): 1.22 (4H, bs, 2 NCH_2_CH_2_CH_2_), 1.49 (4H, bs, 2 NCH_2_CH_2_), 2.59 (6H, s, 2 CH_3_), 3.65 (4H, t, 2 NCH_2_), 7.46–7.65 (8H, m, 2ArH), 13.90 (2H, s, 2SH); ^13^C-NMR (δ ppm): 11.88 (2CH_3_), 20.56 (2NCH_2_CH_2_CH_2_), 27.18 (2NCH_2_CH_2_), 38.79–42.31 (DMSO-d_6_+2NCH_2_CH_2_+2CH_2_), ArC: [119.25 (2C), 126.67 (2C), 131.46 (CH) 147.25 (2CH), 149.74 (2C), 155.01 (2C)], 164.78 (2C=N), 169.27 (2 triazole C=O), MS (ESI): *m/z* (%) 694.22 (M^+^).

*4,4'-(Butane-1,4-diyl)bis(2-((4-(4-bromophenyl)-5-mercapto-4H-1,2,4-triazole-3-yl)methyl)-5-methyl-2H-1,2,4-triazole-3(4H)-one)* (**11a**). Yield 91%, m.p. 296–297 °C. IR (υ, cm^−1^): 2971 (SH), 1697 (triazole C=O), 1,591 (C=N); Anal. Calcd (%) for C_28_H_28_Br_2_N_12_O_2_S_2_: C, 42.65; H, 3.58; N, 21.32. Found: C, 42.70; H, 3.65; N, 21.28; ^1^H-NMR (δ ppm): 1.29 (4H, bs, NCH_2_CH_2_), 2.07 (6H, s, 2CH_3_), 3.36 (4H, bs, 2NCH_2_CH_2_), 4.84 (4H, s, 2NCH_2_C=O), 7.16–7.66 (8H, m, 2ArH), 13.99 (2H, s, 2SH); ^13^C-NMR (δ ppm): 11.04 (2CH_3_), 25.43 (2NCH_2_CH_2_), 38.08–40.59 (DMSO-*d_6_*+2NCH_2_CH_2_+2CH_2_), ArC: [122.79 (2C), 129.56 (2C), 132.14 (CH) 132.22 (2CH), 144.36 (2C), 147.73 (2C)], 152.29 (2C=N), 168.36 (2 triazole C=O), MS (ESI): *m/z* (%) 788.02 (M^+^).

*4,4'-(Hexane-1,6-diyl)bis(2-((4-(4-bromophenyl)-5-mercapto-4H-1,2,4-triazole-3-yl)methyl)-5-methyl-2H-1,2,4-triazole-3(4H)-one)* (**11b**). Yield 89%, m.p. 314–315 °C. IR (υ, cm^−1^): 2969 (SH), 1680 (triazole C=O), 1578 (C=N); Anal. Calcd (%) for C_30_H_32_Br_2_N_12_O_2_S_2_: C, 44.12; H, 3.95; N, 20.58. Found: C, 44.19; H, 34.07; N, 21.64; ^1^H-NMR (δ ppm): 1.20 (4H, bs, 2NCH_2_CH_2_CH_2_), 1.44 (4H, bs, 2 NCH_2_CH_2_), 2.50 (6H, s, 2 CH_3_), 3.71 (4H, t, 2NCH_2_), 7.49–7.69 (8H, m, 2ArH), 13.88 (2H, s, 2SH); ^13^C-NMR (δ ppm): 11.61 (2CH_3_), 20.45 (2NCH_2_CH_2_CH_2_), 27.01 (2NCH_2_CH_2_), 38.80–42.37 (DMSO-*d_6_*+2NCH_2_CH_2_+2CH_2_), ArC: [122.88 (2C), 129.79 (2C), 132.16 (CH) 132.69 (2CH), 144.39 (2C) 147.60 (2C)], 152.41 (2C=N), 167.76 (2 triazole C=O), MS (ESI): *m/z* (%) 816.06 (M^+^).

### 3.7. Antioxidant Activity

**Figure 1:**
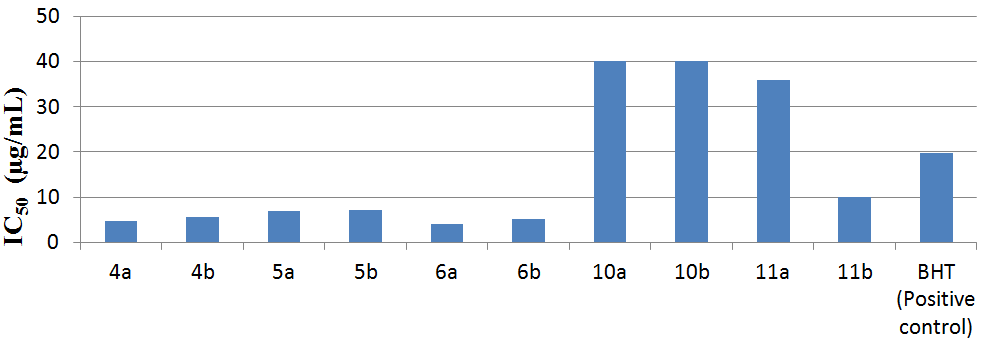
The graphical representation of antioxidant activities compounds.

DPPH assay: The hydrogen atoms or electrons donation ability of the samples was measured from the bleaching of purple coloured methanol solution of DPPH. This spectrophotometric assay uses stable radical 2,2'-diphenylpicrylhydrazyl (DPPH) as a reagent [21,22]. Fifty microliters of various concentrations of the samples in methanol was added to a 0.004% methanol solution of DPPH (5 mL). After a 30 min incubation period at room temperature the absorbance was read against a blank at 517 nm. Inhibition free radical DPPH in percent (*I* %) was calculated in following way: *I* %: (A_blank_ − A_sample_/A_blank_) × 100 where A_blank_ is the absorbance of the control reaction (containing all reagents except the test sample), and A_sample_ is the absorbance of the test compound. Sample concentration providing 50% inhibition (IC_50_) was calculated form the graph plotted inhibition percentage against extract concentration. Tests were carried out in triplicate. Butylated hydroxytoluene (BHT) was used as positive control. The results are shown in the Table 1 and Figure 1.

**Table 1:** IC_50_ values of compounds **4a**,**b**, **5a**,**b**, **6a**,**b**, **10a**,**b** and **11a**,**b**.

Compounds	DPPH IC_50_ (µg/mL) ± 0.5
**4a**	4.7 ± 0.7
**4b**	5.6 ± 0.4
**5a**	7.0 ± 5.7
**5b**	7.1 ± 0.4
**6a**	4.1 ± 0.5
**6b**	5.1 ± 0.3
**10a**	40 ± 2.7
**10b**	40 ± 0.9
**11a**	36± 0.9
**11b**	10 ± 0.7
BHT (Positive control)	19.8 ± 0.5

### 3.8. Antimicrobial Activity

All test microorganisms were obtained from the Hıfzıssıha Institute of Refik Saydam (Ankara, Turkey) and are as follows; Ec; *Escherichia coli* ATCC 25922, Sa; *Staphylococcus aureus* ATCC 25923, Pa; *Pseudomonas aeruginosa* ATCC 27853, Ah; *Acinetobacter haemolyticus* ATCC 19002, Bs; *Bacillus subtilis* ATCC 6633, Pv; *Proteus vulgaris* ATCC 13315, Entc; *Enterobacter cloacea* ATCC 13047, Ef; *Enterococcus faecalis* ATCC 29212, Ca; *Candida albicans* ATCC 60193, Af; *Aspergillus sp*., Fusa; *Fusarium* sp., Rhyso; *Rhizopus* sp. The new compounds were dissolved in dimethylsulphoxide (DMSO) to prepare extract stock solutions of 1,000 μg/mL.

#### Agar Well Diffusion Method

Simple susceptibility screening test using the agar–well diffusion method [23] as adapted earlier was used. Each microorganism was suspended in Brain Heart Infusion (BHI) (Difco, Detroit, MI, USA) broth and diluted to 10^6^ colony forming unit (cfu) per mL. They were “flood–inoculated” onto the surface of BHI agar and Sabouraud Dextrose Agar (SDA, Difco) and then dried. For *C. albicans*, *C. tropicalis*, *Penicillum* spp. and *Aspergillus* spp., SDA was used. Five millimeter diameter wells were cut from the agar using a sterile cork-borer, and 250–5,000 μg/50 μL of the chemical substances were delivered into the wells. The plates were incubated for 18 h at 35 °C. Antimicrobial activity was evaluated by measuring the zone of inhibition against the test organism. Ceftazidime (Fortum) (10 μg) and triflucan (5 μg) were standard drugs. The results are shown in the Table 2.

**Table 2:** Screening for antimicrobial activity of the compounds.

Compounds	Stock Concentration µg/mL	Microorganisms and İnhibition zones (mm)											
		Ec	Sa	Pa	Ah	Bs	Pv	Entc	Ef	Ca	Af	Fusa	Rhyso
**2a**	1000	-	-	-	13	-	-	-	-	13	-	-	-
**3a**	1000	-	-	-	13	-	-	-	-	-	-	-	-
**4a**	1000	-	-	-	12	-	-	-	-	13	-	-	-
**4b**	1000	-	-	-	-	-	-	-	-	11	-	-	-
**5a**	1000	-	-	9	12	-	-	-	-	11	-	-	-
**5b**	1000	-	-	-	-	-	-	12	-	-	-	-	-
**6a**	1000	-	-	-	12	-	11	-	-	11	-	-	-
**6b**	1000	-	-	-	10	-	-	-	-	11	-	-	-
**7a**	1000	-	-	-	12	-	-	-	-	-	-	-	-
**8a**	1000	-	-	-	12	-	-	-	-	-	-	-	-
**8b**	1000	-	-	-	-	-	-	13	-	11	-	-	-
**9a**	1000	-	-	-	12	-	-	12	-	-	-	-	-
**9b**	1000	-	-	-	-	-	-	14	-	-	-	-	-
**10a**	1000	-	-	-	10	-	-	-	-	11	-	-	-
**10b**	1000	-	-	-	-	-	-	11	-	-	-	-	-
Ampicillin			8	5	5	5	11	15	14			-	-
Fortum			45	45	45	20	30	30	35				
Triflucan										25	25		

Ec; *Escherichia coli* ATCC 25922, Sa; *Staphylococcus aureus* ATCC 25923, Pa; *Pseudomonas aeruginosa* ATCC 27853, Ah; *Acinetobacter haemolyticus* ATCC 19002; Bs; *Bacillus subtilis* ATCC 6633, Pv; *Proteus vulgaris* ATCC 13315, Entc; *Enterobacter cloacea* ATCC 13047, Ef; *Enterococcus faecalis* ATCC 29212, Ca; *Candida albicans* ATCC 60193, Af; *Aspergillus* sp., Fusa; *Fusarium* sp., Rhyso; *Rhizopus* sp.

## 4. Conclusions

In this study, a series of new triazole derivatives having carbohydrazide, thiosemicarbazide, thiadiazole and triazole-thiol moieties, respectively, at the 1-position was synthesized, and their antioxidant and antimicrobial activities were evaluated. It was observed that *in vitro* the newly synthesized triazole-thiosemicarbazides **4a**,**b**–**6a**,**b** possess highly potent antioxidant properties and triazole/triazole-thiol derivatives **10a**,**b**–**11a**,**b** possess moderate potent antioxidant properties. Compounds **7a**,**b**, **8a**,**b** and **9a**,**b**, containing triazole-thiadiazole and triazole/triazole-thiol moieties didn’t show antioxidant properties. All newly synthesized compounds were screened for their antibacterial and antifungal activities by the inhibition zones (mm) method. Almost all the synthesized compounds showed significant activity against bacteria, while no compounds showed activity against fungi (*Aspergillus*, *Fusarium*, *Rhizopus*). In particular the 1,2,4-triazole-possessing thiosemicarbazides **4a**,**b**–**6a**,**b** show both antibacterial and antifungal activities.
